# Laboratory and field evaluation of the STANDARD Q and Panbio^™^ SARS-CoV-2 antigen rapid test in Namibia using nasopharyngeal samples

**DOI:** 10.1371/journal.pone.0269329

**Published:** 2022-09-27

**Authors:** Iyaloo Konstantinus, Douglas Chiwara, Emmy-Else Ndevaetela, Victoria Ndarukwa-Phiri, Nathalia! Garus-oas, Ndahafa Frans, Pentikainen Ndumbu, Andreas Shiningavamwe, Gerhard van Rooyen, Ferlin Schiceya, Lindile Hlahla, Pendapala Namundjebo, Ifeoma Ndozi-Okia, Francis Chikuse, Sirak Hailu Bantiewalu, Kapena Tjombonde

**Affiliations:** 1 Department of Research, Namibia Institute of Pathology, Windhoek, Namibia; 2 Department of Quality Assurance, Namibia Institute of Pathology, Windhoek, Namibia; 3 Division of Epidemiology, Ministry of Health and Social Services, Windhoek, Namibia; 4 Department of Molecular Diagnostics, Namibia Institute of Pathology, Windhoek, Namibia; 5 Division of Case Management, Ministry of Health and Social Services, Windhoek, Namibia; 6 World Health Organization Country Office, Windhoek, Namibia; Jahangirnagar University, BANGLADESH

## Abstract

**Background:**

As new SARS-CoV-2 variants of concern emerge, there is a need to scale up testing to minimize transmission of the Coronavirus disease 2019 (COVID-19). Many countries especially those in the developing world continue to struggle with scaling up reverse transcriptase polymerase reaction (RT-PCR) to detect SARS-CoV-2 due to scarcity of resources. Alternatives such as antigen rapid diagnostics tests (Ag-RDTs) may provide a solution to enable countries scale up testing.

**Methods:**

In this study, we evaluated the Panbio^™^ and STANDARD Q Ag-RDTs in the laboratory using 80 COVID-19 RT-PCR confirmed and 80 negative nasopharyngeal swabs. The STANDARD Q was further evaluated in the field on 112 symptomatic and 61 asymptomatic participants.

**Results:**

For the laboratory evaluation, both tests had a sensitivity above 80% (Panbio^™^ = 86% vs STANDARD Q = 88%). The specificity of the Panbio^™^ was 100%, while that of the STANDARD Q was 99%. When evaluated in the field, the STANDARD Q maintained a high specificity of 99%, however the sensitivity was reduced to 56%.

**Conclusion:**

Using Ag-RDTs in low resource settings will be helpful in scaling-up SARS-CoV-2 testing, however, negative results should be confirmed by RT-PCR where possible to rule out COVID-19 infection.

## Introduction

The pandemic caused by Severe Acute Respiratory Syndrome Coronavirus 2 (SARS-CoV-2) identified originally in China has now become a public health concern throughout the world, and now we are dealing with emerging variants of concern (VOCs) of which some are more infectious compared to the founder virus including the current circulating Omicron variant [1–3]. Reverse transcription polymerase chain reaction (RT-PCR) is the laboratory gold standard method to detect SARS-CoV-2 in people with coronavirus disease 2019 (COVID-19). However, this method has its challenges such as long turnaround time, high-cost and requires trained laboratory personnel.

Due to the challenges of using RT-PCR, antigen-rapid diagnostic tests (Ag-RDTs) are now utilized in several countries for epidemiological surveillance and even diagnostic purposes in symptomatic individuals [4]. An observational study in Australian schools found these tests to be a power tool in suppressing the pandemic [5]. In Africa, the low number of COVID-19 cases compared to other continents have been attributed to factors such as low testing due to the high-cost of RT-PCR [6]. Ag-RDTs are less expensive, produce results faster than molecular tests; yielding results in as little as 15 to 30 minutes, and they do not require specialized laboratory techniques [7, 8]. These tests aid in identifying symptomatic COVID-19 positive individuals in a timely manner to prevent further transmission. The WHO recommends Ag-RDTs that meet at least 80% sensitivity and 97% specificity to be utilized in settings where RT-PCR is limited [9]. Namibia received the Panbio^™^ and STANDARD Q Ag-RDTs during the second wave when there was a need to expand COVID-19 testing especially in remote areas which are far from laboratories performing RT-PCR, to shorten the turnaround time. Although these Ag-RDTs have been evaluated in first world countries [10–15], their data remain low in Africa. The Africa-CDC has developed a framework to expand the use of Ag-RDTs including the performance of quality assurance [16, 17].

In the present study, the Panbio^™^ and STANDARD Q were evaluated in detecting SARS-CoV-2 in the laboratory using stored nasopharyngeal samples. The STANDARD Q test was further evaluated at the health care centers using fresh nasopharyngeal samples. The study adds to the much-needed body of evidence on the performance of SARS-CoV-2 Ag-RDTs in sub-Saharan Africa.

## Materials and methods

### Study design and population

The laboratory evaluation was a cross-sectional, retrospective evaluation of the performance of the STANDARD Q (SD Biosensor, Republic of Korea) and Panbio^™^ (Abbott Diagnostic GmbH). Stored nasopharyngeal samples from 80 SARS-CoV-2 positive and 80 SARS-CoV-2 negative RT-PCR confirmed participants were used for this evaluation. The field evaluation was a prospective cross-sectional validation of the STANDARD Q Ag-RDT. Participants 18 years and older seeking SARS-CoV-2 testing were recruited from the Robert Mugabe Clinic and the Katutura Health Center. Participants were either symptomatic with flu-like symptoms, or asymptomatic cases who reported to have been high risk contacts of confirmed cases. The study was explained to the participants by the clinicians and those who chose to participate provided a verbal informed consent. Contact details of the study coordinators were provided in case the participants had complaints or wanted to withdraw from the study. No written informed consent was obtained as this study was conducted during a busy time whereby space and ventilation at these health centers was already a challenge for infectious disease control. This evaluation was approved by the research ethics committee of the Ministry of Health and Social Services (Ref:17/3/3/EEN). Samples were not linked to any personal identifier and results could not be traced back to individual patients.

### RT-PCR and Ag-RDT testing

Nasopharyngeal swabs collected in viral transport medium (VTM) were processed within 24 hours of collection. The viral load was expressed as cycle threshold (CT) value, and a cut of <40 was used. For the laboratory evaluation, nasopharyngeal swabs were stored at -80°C and retrieved for antigen testing within 48 hours by a laboratory personnel according to the manufacturer instructions. Results for STANDARD Q were read strictly within 15–30 minutes, while those of the Panbio^™^ were read in 15 minutes and not beyond 20 minutes as per manufacturer’s instructions. For the field validation using the STANDARD Q Ag-RDT, two nasopharyngeal swabs were collected; one performed by the clinicians on site, and the other swab was transported to the laboratory immediately for testing with RT-PCR.

### Statistical analysis

Sensitivity was calculated as the number of positive samples identified as positive for each Ag-RDT divided by the number of positive samples identified by RT-PCR reference assay. Specificity was calculated as the number of negative samples identified as negative for each Ag-RDT divided by the number of negative samples identified by RT-PCR reference assay. The Positive Predictive Values (PPV) and Negative Predictive Values (NPV) were also calculated. Mann-Whitney U test was computed to compare the differences between two groups using Prism V9 (GraphPad Software).

## Results

### Laboratory performance of the Panbio^™^ and STANDARD Q Ag-RDTs

In total, 160 samples were included of which 80 were RT-PCR positive for COVID-19 with a mean CT value of 25, and 80 were negative (Table 1). The median age was 35 years, and the sample distribution was equal between male (50%) and females (50%).

**Table 1:** Demographics of participants.

	All n = 160	RT-PCR + n = 80	RT-PCR–n = 80
Age, Median [IQR]	35 [26, 48]	33 [26, 45]	37 [26, 49]
Sex, n (%)			
Female	78* (50)	42* (55)	35 (44)
Male	79* (50)	35* (45)	44 (56)
CT value, Mean [Range]		25 [22, 39]	

CT, Cycle threshold; IQR, Interquartile range; RT-PCR, Reverse transcriptase polymerase reaction

*Missing gender data for three samples

The overall sensitivity for the STANDARD Q was 88% (95% CI: 79% to 93%) and the specificity was 99% (95% CI: 93% to 99%), while that of Panbio^™^ was 86% (95% CI: 76% to 91%) and 100% (95% CI: 95% to 100%) respectively (Table 2). The PPV for the STANDARD Q and Panbio^™^ was 99% and 100% respectively, while the NPV was 89% and 87% respectively. When analyzed by CT value, the sensitivity of samples with a CT value ≤25 increased to 97% for both tests (Table 2). For samples with a CT value ≥25, the sensitivity decreased to 76% for STANDARD Q and 71% for the Panbio^™^.

**Table 2:** Determination of the Ag-RDTs sensitivity and specificity.

Ag-RDTs		RT-PCR		Total	Sensitivity	Specificity
STANDARD Q		Positive	Negative		88%	99%
	Positive	70	1	71		
	Negative	10	79	89		
	Total	80	80	160		
CT value ≤25 (n = 42)					97%	
CT value ≥25 (n = 38)					76%	
Panbio^™^		Positive	Negative		86%	100%
	Positive	68	0	68		
	Negative	12	79	91		
	Total	80	79	159		
CT value ≤25 (n = 42)					97%	
CT value ≥25 (n = 38)					71%	

**Fig 1:**
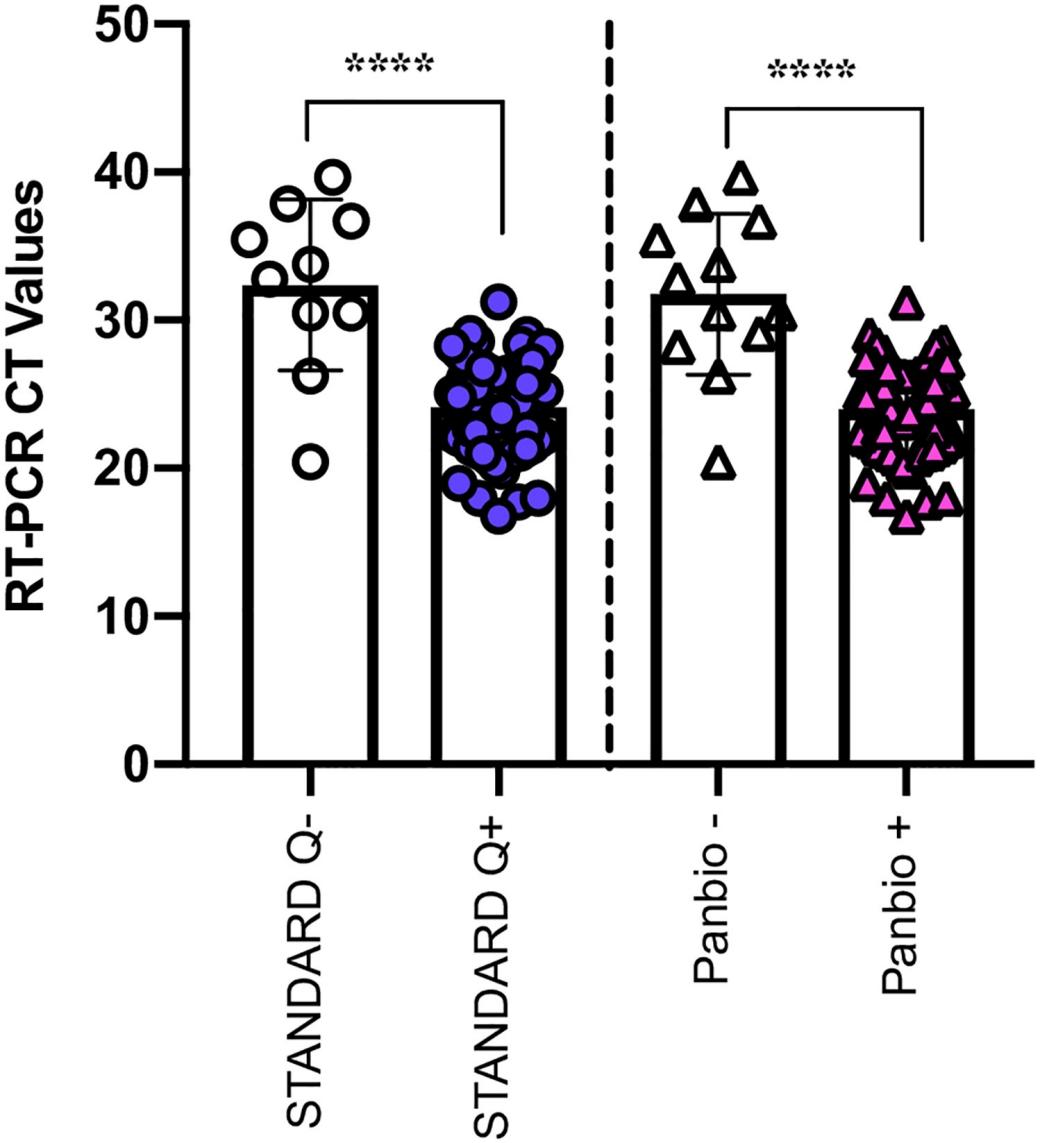
Comparison of CT values in participants who tested positive for SARS-CoV-2 using RT-PCR, grouped according to each Ag-RDT positive and negative results (****p<0.0001).

We then compared CT values grouped according to their Ag-RDT results (Fig 1). STANDARD Q had 70 true positives and 10 false negatives; and Panbio^™^ had 68 true positives and 12 false negatives. As expected, the CT values differed significantly between samples that were either negative or positive by Ag-RDT. For STANDARD Q, the median CT value for the negative samples was 33 compared to a median CT value of 24 in the positive samples. The median CT value for the negative samples using the Panbio^™^ was 32 compared to that of 24 in the positive samples.

### Field performance of the STANDARD Q Ag-RDT

Nasopharyngeal samples from 173 participants were included in the field evaluation study. The demographic and clinical characteristics are shown in Table 3. The median age was 33 years, with an almost equal distribution of females (51%) and males (49%). Of these participants, 65% (n = 112) reported having symptom onset within 5–7 days. Coughing was the most prevalent symptom (37%) followed by a headache (28%), while vomiting was the least common symptom in this cohort. A total of 36 participants tested COVID-19 positive using RT-PCR representing a 21% positivity rate, of which 29 were symptomatic and 7 were asymptomatic.

**Table 3:** Demographics and clinical characteristics of participants.

	All N = 173	RT-PCR + N = 36	RT-PCR–N = 137
Age, Median [IQR]	33 [23, 44]	39 [25, 44]	32 [23, 44]
Sex [n (%)]			
Female	88 (51)	19 (53)	69 (50)
Male	85 (49)	17 (47)	68 (50)
Symptomatic [n (%)]			
No	61 (35)	7 (19)	54 (39)
Yes	112 (65)	29 (81)	83 (61)
Symptoms [n (%)]			
Cough	64 (37)	18 (50)	46 (34)
Fever	12 (7)	2 (6)	10 (7)
Sore throat	35 (20)	8 (22)	27 (20)
Diarrhoea	9 (5)	1 (3)	8 (6)
Loss of smell	16 (9)	5 (14)	11 (8)
Chills	15 (9)	5 (14)	10 (7)
Shortness of breath	5 (3)	0 (0)	5 (4)
Myalgia	12 (7)	5	7 (5)
Vomiting	1 (1)	1	0 (0)
Loss of taste	20 (12)	6	14 (10)
Runny nose	27 (16)	10	17 (12)
Headache	49 (28)	14	35 (26)
Blocked nose	8 (5)	0	8 (6)
Chest pain	11 (6)	0	11 (8)
CT value, Mean [Range]		19 [5, 35]	

CT, Cycle threshold; IQR, Interquartile range; RT-PCR, Reverse transcriptase polymerase reaction

#### STANDARD Q Ag-RDT had a decreased sensitivity in the field

For the field evaluation, only the STANDARD Q Ag-RDT was evaluated due to the unavailability of more Panbio^™^ kits at the time. The STANDARD Q test had a decreased sensitivity of 56% (95% CI: 40% vs 70%) compared to the laboratory sensitivity of 87% (Table 4). Similar to the laboratory evaluation, the specificity in the field was high at 99% (95% CI: 96% to 99%). The PPV was 95%, while the NPV was 90%. The sensitivity of the STANDARD Q test increased to 79% in samples with a CT value ≤**25**.

**Table 4:** Field sensitivity and specificity of the STANDARD-Q Ag-RDT.

Ag-RDT		RT-PCR		Total	Sensitivity	Specificity
STANDARD Q		Positive	Negative		56%	99%
	Positive	20	1	21		
	Negative	16	136	152		
	Total	36	137	173		
STANDARD Q **CT**≤**25**		Positive	Negative		79%	99%
	Positive	19	1	21		
	Negative	5	136	152		
	Total	24	137	173		

**Fig 2:**
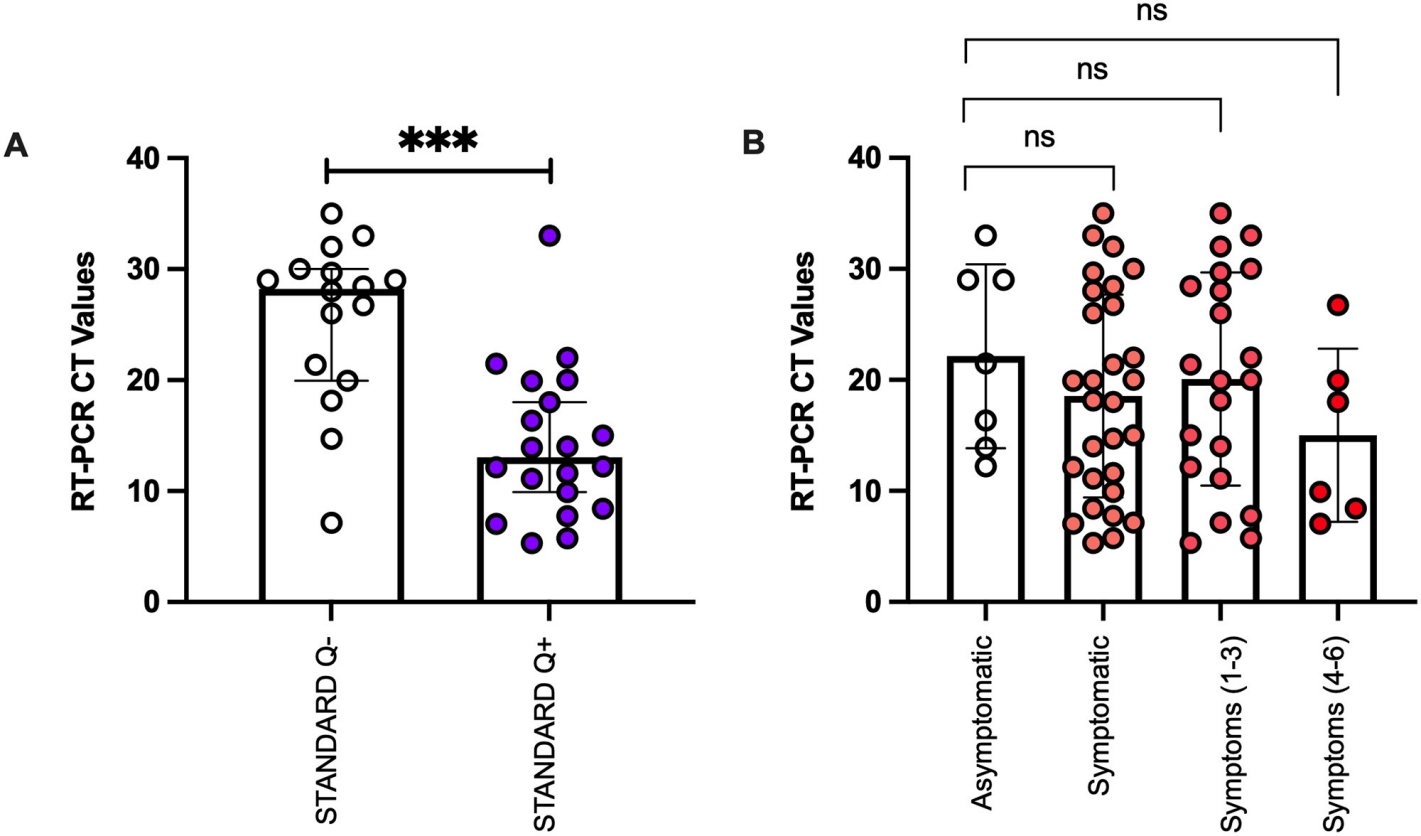
CT values in participants who tested positive for SARS CoV-2 by RT-PCR. (A) Participants grouped according to the STANDARD Q results (***p = 0.0001). (B) Participants were grouped as asymptomatic, all symptomatic, having 1 to 3 symptoms and having 4 to 6 symptoms. P-value was not significant (ns).

We then compared the CT values in participants who were SARS-CoV-2 RT-PCR positive grouped according to their STANDARD Q test result (Fig 2A). Participants with concordant positive results (RT-PCR+ STANDARD Q+) had a significantly lower median CT value of 13 (IQR: 8, 19) compared to those with discordant results (RT-PCR+ STANDARD Q-) with a median CT value of 28 (IQR: 20, 30; P<0.0001). We further grouped these participants according to symptoms; asymptomatic, all symptomatic, those who had 1 to 3 symptoms, and those who had 4–6 symptoms. Although there was a trend towards a high median CT value in asymptomatic compared to symptomatic participants, this was not significant (Fig 2B).

## Discussion

Molecular testing is inherently difficult to scale up as it requires laboratories with specialized equipment and reagents which are costly, and trained laboratory personnel [7, 18]. By the end of 2020, African countries have seen an increase in COVID-19 cases from the second wave of the pandemic compared to the first wave due to an increase infectivity of the new variants of concern such as the Beta, Delta and Omicron [19, 20]. Namibia has not been spared from this, being one of the worst affected African countries during the third wave. Timely and accurate COVID-19 testing is a critical component of surveillance, contact tracing, infection prevention and control and clinical management of COVID-19 cases. Hence, there is a need to continue testing and Ag-RDTs can be utilized in resource limited countries.

In this study, we evaluated two Ag-RDTs, Panbio^™^ and STANDARD Q. The laboratory evaluation showed a high specificity for both tests, with the Panbio^™^ at 100% compared to the STANDARD Q at 98%. In contrast, the sensitivity of the STANDARD Q was slightly higher at 88% compared to that of the Panbio^™^ at 86%. For the field evaluation, only the STANDARD Q Ag-RDT was evaluated due to its availability at the time of conducting the study. Although the STANDARD Q maintained a high specificity of 99% in the field, the test had a reduced sensitivity of 56%. Supporting our findings, field studies in African cohorts on the STANDARD Q conducted in Cameroon and Ghana reported an overall sensitivity of 59% and 64% respectively [21, 22]. The high sensitivity observed in the laboratory might have been due the biased sampling of choosing an equal number of positive and negative samples compared to the randomness in the field of getting a much smaller proportion of individuals testing positive for COVID-19.

Several studies have reported Ag-RDTs to be more sensitive in samples with low CT values as a result of high viral load [23–27]. In the laboratory evaluation, samples with a CT value of 25 and less had an increased sensitivity of 97% for both the STANDARD Q and Panbio^™^, while those with a CT value of 26 above had a sensitivity of 76% and 71% respectively. For the field evaluation, the STANDARD Q sensitivity increased by 79% in samples with a CT value of 25 and less. One study reported sensitivity of the Panbio^™^ and STANDARD Q to reach 100% for samples with a CT value below 20, decreasing at 41% and 52% respectively for those samples with a CT value between 25–30 [27]. Other studies reported the sensitivity of these two Ag-RDTs to increase above 80% when the CT value was <25 [18, 21, 25, 26, 28–31].

Limitations of this study includes the lack of data on symptoms for participants included in the laboratory evaluation. For the field evaluation, credibility of the symptom onset of the participants could not be determined if it was within 5–7 days. This is important because these tests have been reported to be more reliable in detecting SARS-CoV-2 infection within the first 7 days after the onset of symptoms. Therefore, they can miss individuals who are in the very early stage of infection (presymptomatic stage) and those who are in the late stage with decreased viral replication. Moreover, the laboratory evaluation used one nasopharyngeal swab for both the RT-PCR and antigen testing unlike the field evaluation which collected two nasopharyngeal samples and this biased approach might have contributed to the high sensitivity observed in the laboratory. The laboratory evaluation was also conducted in a well-lit controlled environment by a trained technician as opposed to the reality in the field.

In conclusion, these results add to the body of evidence that Ag-RDTs are useful and may be utilized to scale up testing in symptomatic individuals to reduce viral transmission in settings where RT-PCR is a challenge.

## Supporting information

S1 Dataset (XLSX)
